# Ultrafast Opto‐Electronic and Thermal Tuning of Third‐Harmonic Generation in a Graphene Field Effect Transistor

**DOI:** 10.1002/advs.202401840

**Published:** 2024-06-18

**Authors:** Omid Ghaebi, Sebastian Klimmer, Nele Tornow, Niels Buijssen, Takashi Taniguchi, Kenji Watanabe, Andrea Tomadin, Habib Rostami, Giancarlo Soavi

**Affiliations:** ^1^ Institute of Solid State Physics Friedrich Schiller University Jena 07743 Jena Germany; ^2^ ARC Centre of Excellence for Transformative Meta‐Optical Systems Department of Electronic Materials Engineering Research School of Physics The Australian National University Canberra ACT 2601 Australia; ^3^ Research Center for Materials Nanoarchitectonics National Institute for Materials Science 1‐1 Namiki Tsukuba 305‐0044 Japan; ^4^ Research Center for Electronic and Optical Materials National Institute for Materials Science 1‐1 Namiki Tsukuba 305‐0044 Japan; ^5^ Dipartimento di Fisica Università di Pisa Largo Bruno Pontecorvo 3 Pisa 56127 Italy; ^6^ Department of Physics University of Bath Claverton Down Bath BA2 7AY UK; ^7^ Abbe Center of Photonics Friedrich Schiller University Jena 07743 Jena Germany

**Keywords:** all‐optical THG modulation, electrically tunable THG, graphene, nonlinear optics

## Abstract

Graphene is a unique platform for tunable opto‐electronic applications thanks to its linear band dispersion, which allows electrical control of resonant light‐matter interactions. Tuning the nonlinear optical response of graphene is possible both electrically and in an all‐optical fashion, but each approach involves a trade‐off between speed and modulation depth. Here, lattice temperature, electron doping, and all‐optical tuning of third‐harmonic generation are combined in a hexagonal boron nitride‐encapsulated graphene opto‐electronic device and demonstrate up to 85% modulation depth along with gate‐tunable ultrafast dynamics. These results arise from the dynamic changes in the transient electronic temperature combined with Pauli blocking induced by the out‐of‐equilibrium chemical potential. The work provides a detailed description of the transient nonlinear optical and electronic response of graphene, which is crucial for the design of nanoscale and ultrafast optical modulators, detectors, and frequency converters.

## Graphical abstract

This work demonstrates thermal, electrical, and all‐optical tuning of the nonlinear optical response of hBN encapsulated monolayer graphene, considering the interplay between electronic temperature, Pauli blocking, and electron dephasing. The device displays electrostatic third‐harmonic (TH) modulation for both electrons and holes, and ultrafast all‐optical TH modulation with close to 90% modulation depth.

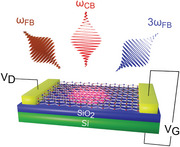

## 1 Introduction

2D materials are ideal candidates for nonlinear optical applications at the nanoscale,^[^ ^1^ ^]^ as they enable ultra‐broadband optical parametric amplification,^[^ ^2^ ^]^ spontaneous parametric down‐conversion,^[^ ^3^ ^]^ electrical, and all‐optical tuning of the second harmonic (SH)^[^ ^4^, ^5^, ^6^, ^7^ ^]^ and third harmonic (TH) generation,^[^ ^5^, ^8^, ^9^ ^]^ giant efficiencies of THz high harmonic generation,^[^ ^10^ ^]^ and applications in integrated nonlinear opto‐electronic devices such as gas sensors,^[^ ^11^ ^]^ logic gates,^[^ ^12^, ^13^ ^]^ and valleytronics.^[^ ^14^, ^15^ ^]^

Text within the family of 2D materials, graphene arguably shows the most intriguing nonlinear response. Being centrosymmetric, the first nonlinear term in its polarization is the third‐order susceptibility χ^(3)^. While few experimental studies have observed second‐harmonic generation (SHG) due to breaking of symmetry at an interface,^[^ ^16^, ^17^ ^]^ in‐plane electric fields and currents^[^ ^18^, ^19^ ^]^ or from the electric quadrupole response.^[^ ^20^ ^]^ the vast majority of nonlinear optical experiments on graphene have focused on χ^(3)^ processes such as four‐wave mixing (FWM),^[^ ^21^ ^]^ third‐harmonic generation (THG),^[^ ^8^, ^9^, ^22^, ^23^, ^24^ ^]^ and saturable absorption.^[^ ^25^, ^26^, ^27^ ^]^ In particular, THG and FWM have recently gained increasing attention following the demonstration of their electrical^[^ ^8^, ^9^, ^21^ ^]^ and all‐optical^[^ ^5^ ^]^ modulation, which provide a route towards ultrafast nanoscale frequency converters and a powerful method to probe ultrafast hot electron dynamics. The electrical tunability of THG in graphene has been widely explored,^[^ ^8^, ^9^, ^21^, ^22^ ^]^ whereas ultrafast all‐optical modulation and the interplay of lattice (*T*_L_) and electron temperatures (*T*_e_) in high‐quality hexagonal boron nitride (hBN)‐encapsulated graphene samples are scarcely studied.

In this work, we provide a detailed experimental and theoretical study of ultrafast thermal and opto‐electronic modulation of THG in a high‐quality and gate‐tunable hBN/graphene/hBN field effect transistor (FET). Encapsulation of graphene in hBN is widely used to achieve a higher sample quality, and to engineer the *T*_e_ via out of plane heat transfer.^[^ ^28^, ^29^ ^]^ In our experiments, we further use hBN encapsulation to reduce the intrinsic doping of graphene, and with this, we demonstrate for the first time ambipolar gate tunable THG, as discussed in the following. Our scheme for opto‐electronic THG modulation can be briefly summarized as follows. We irradiate graphene with two pulses: a fundamental beam (FB) and a control beam (CB). The FB is responsible for inducing the parametric THG process ($\omega_{\mathrm{FB}}\to 3\omega_{\mathrm{FB}}$) while the CB controls the TH efficiency via tuning of *T*_e_ and Pauli blocking. We point out from the very start that the FB affects *T*_e_ and Pauli blocking as well, due to its large fluence (comparable to the CB), necessary to generate a sizable TH. Furthermore, electrical doping by means of external gates enables the system to modulate the competition between *T*_e_ and Pauli blocking mechanisms and to tune the TH ultrafast recombination dynamics. Thus, by combining electrical and all‐optical control of *T*_e_ and Fermi Energy (*E*_F_), we achieve active modulation of THG in graphene with the following main results. First, experiments on hBN‐encapsulated samples allow to show that the electrical modulation of THG in graphene is symmetric for electrons and holes within the Dirac cone. This is the nonlinear optical analog of the electronic ambipolar behavior of FETs, which was absent in previous studies.^[^ ^8^, ^21^ ^]^ Further, we observe up to 300% modulation in the THG intensity by tuning *T*_L_ from 295 to 33 K. Second, we show that electrical doping can be used to actively control the recombination dynamics of the TH signal arising from phase‐space quenching of the scattering between hot electrons and optical phonons.^[^ ^30^ ^]^ Third, we shed light on the physical origin of the ultrafast TH modulation and the interplay of hot electrons and Pauli blocking. Finally, with our nonlinear opto‐electronic device, we achieve a TH modulation depth of ≈85% at *E*_F_ = 300 meV and peak fluence of 200 µJ cm^−2^, namely a two orders of magnitude enhancement in the modulation efficiency (i.e., modulation depth per unit of fluence) compared to previous reports.^[^ ^5^ ^]^ This is possible thanks to mid‐IR excitation and active control of *E*_F_ and *T*_L_ and thus it further clarifies that a deeper understanding of the ultrafast and nonlinear opto‐electronic response of graphene is paramount for the design and optimization of nanoscale ultrafast devices, such as optical modulators, detectors, and frequency converters.

## 2 Ambipolar Gate‐Tunable THG

**Figure 1:**
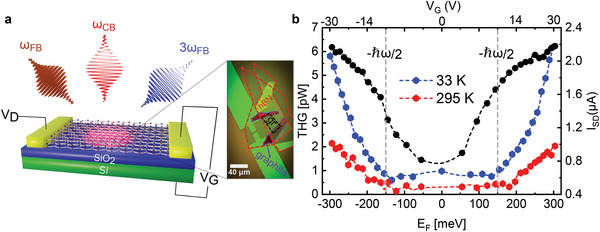
Opto‐electronic modulation of THG in a graphene FET. a) Sketch and microscope optical image of the device. Monolayer graphene is encapsulated between two hBN flakes. *V*_G_, *V*_D_, ω_FB_, and ω_CB_ represent the gate‐source voltage, source‐drain voltage, fundamental beam, and control beam, respectively. b) THG as a function of *E*_F_ (bottom x‐axis) and *V*_*G*_ (top x‐axis) at lattice temperatures of *T*_L_ = 295 K (red curve) and *T*_L_ = 30 K (blue curve). The black curve is the drain current (*I*_*D*_) as a function of *E*_F_ and *V*_G_ at the drain voltage of *V*_D_ = 1 mV.

Opto‐electronic (i.e., optical and electrical) modulation of THG is performed on a back‐gated FET based on a single layer graphene encapsulated in two ≈10 nm thick hBN layers (**Figure** **1**a). The device was prepared by mechanical exfoliation and dry transfer, following the approach described in ref. [31] (see Sections S1 and S2, Supporting Information for details on sample fabrication and characterization). For the THG measurements, we used two synchronized laser pulses at a repetition rate of 76 MHz, photon energies of 0.32 eV (3900 nm) and 1.2 eV (1030 nm) and pulse duration of ≈150 fs/110 fs for the FB/CB, respectively. The spot‐sizes of the focused FB and CB have been measured using the razor blade technique^[^ ^32^ ^]^ and they are ≈6.7 and ≈2.2 µm, respectively (see Section S3, Supporting Information).

First, we measured gate‐tunable THG with a “static” procedure (i.e., without CB). We irradiate our device with the FB (130 µJ cm^−2^) and collect the TH power for different values of the applied *V*_G_ in the range −30 to 30 V, corresponding to values of the *E*_F_ in the range −300 to 300 meV (see Section S2, Supporting Information for the calculation of *E*_F_) and for different *T*_L_. We stress that, under these experimental conditions, the TH intensity from the hBN encapsulant is negligible (see Section S4, Supporting Information). The experimental data (Figure 1b) show a modulation factor of ≈4 when *T*_L_ = 295 K and the *E*_F_ is tuned from ≈50 to 300 meV. This gate‐tunable TH modulation is due to the crossing of multi‐photon resonances in the Dirac cone, as largely discussed in refs. [8, 9]. Once the *T*_L_ is decreased to 33 K, the modulation factor in the same *E*_F_ range increases to ≈9. Comparing the two curves at different temperatures, we observe an enhancement of the TH power while reducing *T*_*L*_ of ≈1.5 and ≈3 at *E*_F_ = 50 meV and *E*_F_ = 300 meV, respectively. The origin of this remarkable enhancement of THG with *T*_L_ is manifold. Our theoretical analysis reproduces this effect, on a smaller magnitude, solely based on the different electron distribution achieved when samples with different *T*_L_ are irradiated by the same FB. This is an indirect effect of *T*_L_ on THG, due to the different dynamics experienced by electrons on a statistical level. However, we assume that a contribution to the observed TH enhancement arises also from a direct effect of temperature at the level of single‐particle, coherent evolution during the FB pulse duration. Such an effect can be attributed to the temperature‐dependent electron scattering rates (or electron spectral broadening) with impurities, defects and phonons (see Section S6, Supporting Information). Although our numerical calculations support this argument, a solid determination of the scattering rates at different temperatures would require a much larger amount of data sets that is outside the scope of this work.

The absence of sharp peaks in the data reported in Figure 1b is a clear indication of the high *T*_e_ reached during the experiments,^[^ ^8^, ^9^ ^]^ as we discuss in detail in the Section S5 (Supporting Information). Since *T*_e_ is a function of *E*_F_ and varies dramatically over the pulse duration, we cannot assign a single value of *T*_e_ to the points in Figure 1b. However, if we consider, e.g., *T*_L_ = 33 K and *E*_F_ = 50 meV, our calculations show that a *T*_e_ > 1400 K is achieved by the electron distribution for over 200 fs, at the FB peak fluence of 130 µJ cm^−2^ (see also Section S5, Supporting Information). We point out that we observe gate‐tunable THG for both positive and negative values of the *E*_F_, indicating that the TH enhancement at multi‐photon resonances can be achieved for both n‐ and p‐doping, i.e., in the conduction and valence band of the Dirac cone, qualitatively preserving the electron‐hole symmetry of the phenomenon to a remarkable degree.

Finally, the results reported in Figure 1b allow us to estimate the χ^(3)^ of graphene at different values of *E*_F_, at the FB photon energy of 0.32 eV by using the two following equations:^[^ ^22^ ^]^

(1) $$P\left(\omega_{i,o}\right)=\frac{1}{8}{\left(\frac{\pi }{\ln\;2}\right)}^{3/2}f\tau W^{2}n_{\omega_{i,o}}\varepsilon_{0}c\frac{|E\left(\omega_{i,o}\right){|}^{2}}{2}$$

(2) $$E\left(\omega_{o}\right)=\frac{1}{4}\frac{i\omega_{i}}{2\pi c}\chi_{\exp}^{\left(3\right)}d_{\mathrm{gr}}E^{3}\left(\omega_{i}\right)$$

where *P*(ω_*i*, *o*_), *E*(ω_*i*, *o*_) are the input/generated TH power and electric field and f,τ, $n_{\omega_{i,o}}$ are the repetition rate, pulse duration, and refractive index, respectively. The input/TH electric fields can be extracted from Equation (1) and then the χ^(3)^ value can be calculated using Equation (2). *d*_gr_ = 0.3 nm is the thickness of monolayer graphene. Considering the losses of the setup (see Section S3, Supporting Information) and *T*_L_ = 33 K we obtain χ^(3)^ ≈ 2 × 10^−15^ m^2^ V^−2^ for *E*_F_ ≈ 300 meV and ≈ 8 × 10^−16^ m^2^ V^−2^ for *E*_F_ ≈ 0 meV, in agreement with ref. [22] where a χ^(3)^ ≈ 6 × 10^−16^ m^2^ V^−2^ was reported for pristine graphene at a fundamental photon energy of 0.225 eV and *E*_F_ = 390 meV.

## 3 Ultrafast Opto‐Electronic TH Modulation

**Figure 2:**
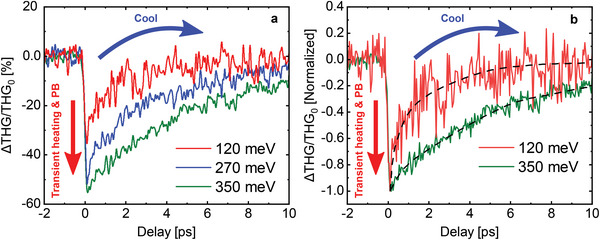
All‐optical modulation of THG and gate tunable dynamics. a) Ratio $\frac{\Delta THG}{THG_{0}}$, (defined in equation (3)), for different values of *E*_F_. *THG*_0_ has been measured at −2 ps. The interplay of transient heating and PB (Pauli blocking) on electrons will occur when CB and FB pulses are spatially and temporally synchronized and subsequent cooling occurs via electron–electron and electron–phonon scattering. b) Normalized $\frac{\Delta THG}{THG_{0}}$ for *E*_F_ = 120 meV and *E*_F_ = 350 meV.

Next, we shift our attention to time‐resolved and all‐optical TH modulation. We initially fix the CB and FB fluence at 170 and 110 µJ cm^−2^, respectively, and scan their relative delay for different values of *E*_F_ in the range 0 to 390 meV. We remark that this range of *E*_*F*_ overlaps the region defined by the lower threshold *E*_F_ > ℏω/2, where absorption of the FB, at zero temperature, is forbidden by Pauli blocking. However, we do not see an abrupt drop‐off of the measured signal when the (*E*_F_) exceeds such threshold. The reason is that the finite temperature in our samples ensures that a residual absorption is always present. Even a small initial absorption produces a rapid temperature increase, which broadens the electron distribution in the energy space and relaxes the condition for Pauli blocking. To mitigate the effect of diminished absorption, in the following, we discuss the behavior of the measured signal divided by the signal before the pump is applied, thus “normalizing‐out” the most trivial part of the Pauli blocking. We point out, however, that other non‐trivial thresholds appear in the THG as the Fermi energy crosses multiples of the FB frequency.^[^ ^8^, ^9^ ^]^ **Figure** **2** shows the experimental results for the ratio Δ*THG*/*THG*_0_, where

(3) $$\Delta THG\left(\tau \right)=THG\left(\tau \right)-THG_{0}\;$$

*THG*(τ) is the measured signal as a function of delay τ, and *THG*_0_ is the reference TH signal measured in the absence of the CB, that we measure at a negative delay τ = −2 ps. As expected, the signal features a sharp peak when the FB overlaps with the CB, i.e., when both beams excite the electron system, followed by a “relaxation” stage converging to a zero signal, which represents the recovery of the system from the excitation due to the CB. At large delays, the effect of the CB vanishes and the TH signal recovers to its reference value *THG*_0_.

The process of electron relaxation in graphene after excitation from an ultrashort pulse has been discussed at length in the literature,^[^ ^33^, ^34^, ^35^, ^36^, ^37^, ^38^ ^]^ and in summary, involves: i) an initial stage dominated by electron–electron interactions where the photoexcited electron system achieves thermalization at a temperature much higher than the initial (lattice) temperature, possibly with inter‐band processes associated to Auger recombination and carrier multiplication; ii) a first cooling stage dominated by the emission of optical phonons where both the electron temperature and the photoexcited density decreases; iii) a second, slower cooling stage, where the hot optical phonons thermalize with the acoustic phonons of the lattice, possibly with the intervention of “supercollision” processes, and the unperturbed initial state is finally recovered. We remark again that the FB, due to its fluence, strongly perturbs the electron system, such that, even several ps after the CB, the TH signal cannot be considered as the response of an electron system at equilibrium with the lattice.

From the data in Figure 2, we also notice that the rate of relaxation diminishes as the Fermi energy is increased. We recognize this effect as the quenching of optical phonon emission in the first cooling stage, due to the reduction of the available phase‐space for electronic transitions, which was recently discussed in ref. [30]. In other words, due to Pauli blocking, photoexcited electrons at energy *E* can only emit a phonon of energy ℏω_ph_, if states are available at energy *E* − ℏω_ph_. As the Fermi energy is increased, and approaches the photoexcitation energy, this condition is harder and harder to satisfy, even at large temperature where the electron distribution is broadened. It is interesting that this phase‐space effect does not only affect the differential transmission of the electron system, as demonstrated in ref. [30], but emerges in the measurement of the TH as well. This observation highlights how consequential it is to be able to tune the electron density by electrical doping in a graphene‐based optoelectronic device, thus exerting a certain degree of control on both its linear and non‐linear optical response.

**Figure 3:**
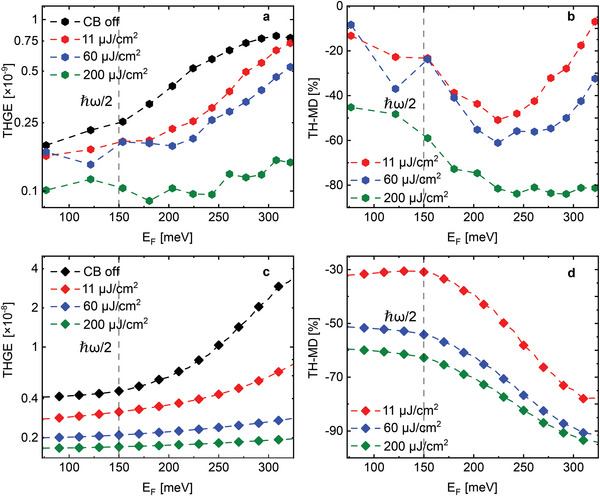
Influence of *E*_F_ and CB peak fluence on THGE and TH‐MD. a,b) Experimental THGE and TH‐MD (defined in Equation (4)) as a function of *E*_F_, for different values of the CB peak fluence reported in the legend. c,d) Theoretical THGE and TH‐MD calculated using the experimental values of the incident peak fluences of CB and FB.

Finally, we explore the dependence of THG on the state of the electron system before the FB, by changing the fluence of the CB. In **Figure** **3**a we plot the THG efficiency (THGE) and in Figure 3b the third harmonic modulation depth (TH‐MD), defined as

(4) $$\mathrm{THGE}=\frac{P_{TH}}{P_{FB}}\;,\;\mathrm{TH}-\mathrm{MD}=\frac{\Delta P_{TH}}{P_{TH_{0}}}\;$$

respectively, where Δ*P*_*TH*_ is the difference in the TH power (*P*_*TH*_) with and without ($P_{TH_{0}}$) the CB, and *P*_*FB*_ is the power of the fundamental beam. The data are shown as a function of *E*_F_ and for different values of the incident CB fluence. In all the experimental graphs, the data are extracted at zero time delay between the FB and CB.

When the CB is off (black symbols in Figure 3a), we obtain a similar result reported in Figure 1b, namely an increase of the THGE when ℏω < 2*E*_F_. The same trend can be observed when we switch‐on the CB, but in this case, the modulation factor with respect to *E*_F_ is reduced. When the CB fluence reaches 200 µJ cm^−2^ (green symbols) the modulation factor is close to zero and the THGE is almost constant over the measured range of *E*_F_.

The TH‐MD is in the range ≈7 to 85% for CB peak fluences of 11 to 200 µJ cm^−2^. Interestingly, we obtain a maximum TH‐MD of 85% for *E*_F_ = 300 meV and peak fluence of 200 µJ cm^−2^. This exceeds by far the results of ref. [5], where a similar TH‐MD of 90% was obtained for a CB peak fluence of 25 mJ cm^−2^. Two features of the data deserve to be highlighted: i) tuning *E*_F_ plays a huge role in the TH‐MD; ii) for all values of *E*_F_ we observe a negative TH‐MD.

Figure 3c,d show our theoretical calculations for the THGE and TH‐MD, respectively, obtained by means of the model discussed in the following section. The overall agreement between theory and experiment is satisfactory, albeit with two shortcomings. The first is an overall factor in the magnitude of the signal, which can easily be traced back to an incomplete determination of some fitting parameters, such as the attenuation of the signal in the detection apparatus, or the electron scattering rates in the theoretical expression of the THG (see Section S6, Supporting Information). The second is the missing ramp‐up of the TH‐MD at *E*_F_ ≳ 250 meV. We find this discrepancy similar to what was reported in ref. [38] in the context of the quenching of the optical phonon‐emission by Pauli blocking and attribute it to the theoretical model missing a Fermi‐energy‐dependent effect that enhances electron recombination. In any case, these two shortcomings do not hinder our understanding of the main feature which we are concerned with in the present work, namely the all‐optical switching of the TH signal. The theoretical results fully support our picture that the variations of the measured signal are due to the effect of the CB on the electron distribution before the sample is irradiated by FB.

## 4 Theory of Ultrafast Opto‐Electronic THG Modulation

### 4.1 THG Efficiency for Photoexcited Electrons

In order to rationalize our experimental results, we need to extend the theoretical treatment of the THG^[^ ^8^, ^9^ ^]^ to take into account the specific role that the CB plays in the dynamics of the electron system. Indeed, the key issue of the CB‐FB protocol used in our experimental procedure is that the increase of *T*_*e*_, due to the heat delivered by the CB, is inextricably linked to the production of a photoexcited electron density (δ*n*_e_), i.e., an excess electron (hole) density in the conduction (valence) band. We emphasize that such an excess carrier density is larger than the density that appears in an equilibrium system when the temperature is increased, purely due to the broadening of the Fermi‐Dirac distribution across the Dirac point. Mathematically, δ*n*_e_ results in the splitting of the chemical potential (μ) into two different chemical potentials μ_C_, μ_V_ for the electrons in conduction and valence bands, respectively, also known as “quasi‐Fermi energies”. We emphasize that the proper *E*_F_, an equilibrium quantity that corresponds to the value of the chemical potential at vanishing temperature, is in a one‐to‐one correspondence to the electron density due to doping, and does not change due to process of inter‐band photoexcitation.

Following refs. [8, 9], it is convenient to factor Equation (4) for THGE as

(5) $$THGE=\frac{n_{b}}{n_{t}^{3}{\left(n_{t}+n_{b}\right)}^{2}}{\left(\frac{I_{\mathrm{FB}}}{W_{0}}\right)}^{2}{\left|S\left(\omega_{\mathrm{FB}}+i\Gamma_{e},\mu_{C},\mu_{V},T_{e}\right)\right|}^{2}\;$$

where *n*_t_, *n*_b_ are the refractive indices of the top and bottom substrates, respectively, and the quantity *W*_0_ = 10^12^ W m^−2^ is introduced to render the expression dimensionless. Finally, the factor *S* is the TH conductivity, which depends on the frequency ω_FB_ of the FB pulse and on the thermodynamic variables of the photoexcited electron system, i.e., *T*_*e*_ and the two chemical potentials μ_C_ and μ_V_. The expression for the TH conductivity at zero temperature (*T*_e_ = 0), in the absence of photoexcited density (δ*n*_e_ = 0, i.e., μ_C_ = μ_C_ = ε_F_), was given in ref. [39] in a fully analytical form, and reads

(6) $$\begin{array}{c} S\left(\hbar \omega_{\mathrm{FB}}+i\Gamma_{e},E_{F}\right)=K\left(E_{F}\right)\frac{17G(X/2)-64G(X)+45G(3X/2)}{X^{4}}\; \end{array}$$

in terms of the dimensionless function *G*(*X*) = ln [(1 + *X*)/(1 − *X*)]. The parameter *K* is a dimensionless constant given by

(7) $$K\left(E_{F}\right)=\frac{W_{0}}{2\varepsilon_{0}^{2}c^{2}}\frac{e^{4}\hbar v_{F}^{2}}{192\pi E_{F}^{4}}\;$$

Finally, the dimensionless quantity *X* = (ℏω_FB_ + *i*Γ_e_)/|*E*_F_| in Equation (6) is the energy of the FB photons, rescaled by *E*_F_, and includes an imaginary contribution due to the effective electron scattering rate Γ_e_. The expression of Γ_e_ depends on the precise scattering channel responsible for the finite electron mobility, such as charged impurities, phonon, defects etc., and it might depend on the electron doping as well as the electron and lattice temperatures (see Section S6, Supporting Informarion).

To obtain the expression of the TH conductivity of the photoexcited electron gas, we now apply a well‐known algebraic trick due to Maldague,^[^ ^40^ ^]^ as detailed in ref. [41] for the linear polarization function (i.e., the Lindhard function). This approach allows us to calculate the desired quantity numerically, using an energy‐integral over the analytical expression given in Equation (6):

(8) $$\begin{array}{cc} & S(\hbar \omega_{\mathrm{FB}}+i\Gamma_{e},\mu_{C},\mu_{V},T_{e})\\ & \;=\frac{1}{4k_{B}T_{e}}\int_{0}^{\infty }dE\left\{\frac{S\left(\hbar \omega_{\mathrm{FB}}+i\Gamma_{e},E\right)}{\cosh^{2}\left(\frac{E-\mu_{C}}{2k_{B}T_{e}}\right)}+\frac{S\left(\hbar \omega_{\mathrm{FB}}+i\Gamma_{e},E\right)}{\cosh^{2}\left(\frac{E+\mu_{V}}{2k_{B}T_{e}}\right)}\right\}\\ & \;-\left\{\frac{1}{e^{-\mu_{C}/k_{B}T_{e}}+1}-\frac{1}{e^{-\mu_{V}/k_{B}T_{e}}+1}\right\}S\left(\hbar \omega_{\mathrm{FB}}+i\Gamma_{e},E_{F}\to 0\right)\; \end{array}$$

The standard mathematical expression that relates the μ_V_, μ_C_, the δ*n*_e_, and *E*_F_ can be found e.g., in ref. [8].

**Figure 4:**
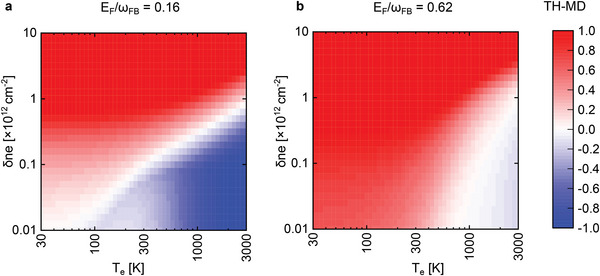
TH‐MD of the photoexcited electron system. The calculated value of the TH‐MD as a function of *T*_*e*_ and δ*n*_e_ at fixed *E*_F_ equal to (a) 0.16 and (b) 0.62 in units of the FB frequency ω_FB_. At a fixed *T*_*e*_, increasing the value of δ*n*_*e*_ will enhance the THG signal, while at a fixed δ*n*_*e*_, increasing *T*_*e*_ will reduce THG intensity. Both *T*_*e*_ and δ*n*_*e*_ can be controlled by the incident laser power absorption.

To better illustrate the dependence of the THG on a variation of electron temperature and photoexcited electron density, in **Figure** **4**, we show the profile of the TH‐MD (defined in Equation (4)), with respect to a reference state with *T*_e_ = *T*_L_ and vanishing δ*n*_e_. As expected from the equilibrium results,^[^ ^39^ ^]^ increasing the *T*_*e*_ generally lowers the value of the *P*_*TH*_ (i.e., negative TH‐MD). Increasing the δ*n*_e_, on the contrary, increases the *P*_*TH*_, as can also be expected from the doping‐dependence known from the equilibrium results.^[^ ^39^ ^]^ In other words, δ*n*_e_ can be seen as a quasi‐equilibrium electron‐ and hole‐doping in conduction and valence band, respectively. It follows that the CB can affect the THG in two competing ways because it produces a *T*_*e*_ increase that is necessarily coupled to the production of δ*n*_e_. It is then necessary to know the precise relation between *T*_e_(*t*) and δ*n*_e_(*t*) in time to predict the THG following a given CB. To this end, we resort to the solution of a model dynamics, based on a simple rate‐equation approach, which we outline in the following section.

Before we discuss our dynamical model, we remark that the procedure that leads to Equation (8) cannot be applied to arbitrary non‐equilibrium states of the electron system, but assumes that the carriers in the two bands are thermalized to the same *T*_e_, although it allows for two different μ_V_ and μ_C_. Mathematically, this means that the electron (hole) distribution in the conduction (valence) band is given by a Fermi‐Dirac function of the form

(9) $$\begin{array}{c} f_{e,h}\left(E,\mu_{C,V}\left(t\right),T_{e}\left(t\right)\right)=\frac{1}{e^{\left(E\pm \mu_{C,V}\left(t\right)\right)/k_{B}T_{e}\left(t\right)}+1}\; \end{array}$$

where the carrier energy *E* is measured from the Dirac point.The quasi‐equilibrium assumption of Equation (9) then holds if the system's dynamics is coarse‐grained on a time‐scale longer than the electron thermalization time‐scale, which has been shown to be shorter than ≈20 fs in graphene.^[^ ^35^ ^]^ The dynamical model that we adopt here is fully consistent with this limitation.

### 4.2 Model Dynamics of Photoexcited Electrons

To model the dynamics of photoexcited electrons, we adopt a rate‐equation approach that describes: i) electron heating due to the laser beams; ii) energy exchange between electrons and optical phonons, due to emission and absorption processes; iii) optical phonon relaxation to the lattice equilibrium temperature (see e.g., ref. [38] and references therein). The variables of interest are the *T*_e_(*t*), δ*n*_e_(*t*), and the occupation of the optical phonon modes around the center of the Brillouin zone (Γ point) and the valleys (*K* points), with frequencies $\omega_{\Gamma }$ and ω_*K*_, respectively.

The time‐derivative of the *T*_*e*_ is given by the net absorbed power divided by the heat capacity

(10) $$\frac{dT_{e}\left(t\right)}{dt}=\frac{P\left(t\right)-R_{\Gamma }\left(t\right)\hbar \omega_{\Gamma }-R_{K}\left(t\right)\hbar \omega_{K}}{c_{e}\left(t\right)+c_{h}\left(t\right)}\;$$

where $P\left(t\right)=P_{\mathrm{FB}}\left(t\right)+P_{\mathrm{CB}}\left(t\right)$ is the average power absorbed per unit area, *c*_e, h_(*t*) are the electron and hole heat capacity per unit area, and *R*_Γ, *K*_(*t*) are the net phonon emission and absorption rates. The expressions for the electron absorbance (that relates the absorbed to the incident power in the linear regime) and the heat capacity can be found e.g., in ref. [8]. Here, we calculate the heat capacity as the sum of the electron and hole contribution, taken into account independently, because inter‐band recombination processes are much slower than thermalization, and thus do not contribute to the temperature adjustment, which is mathematically described by the heat capacity coefficient. The phonon rates follow from a standard Boltzmann formula that can be found e.g., in ref. [38]. We remark that the coefficients discussed above depend on the electron distribution and phonon occupation, and must thus be calculated dynamically in time as the system evolves. Notwithstanding its simple appearance, Equation (10) is a strongly non‐linear equation of motion.

The time derivative of the δ*n*_e_ is given by the number of photons absorbed minus the number of phonons emitted by interband transitions, per unit time and area

(11) $$\frac{d\delta n_{e}\left(t\right)}{dt}=\frac{P_{\mathrm{CB}}\left(t\right)}{\hbar \omega_{\mathrm{CB}}}+\frac{P_{\mathrm{FB}}\left(t\right)}{\hbar \omega_{\mathrm{FB}}}-R_{\Gamma ,\mathrm{inter}}\left(t\right)-R_{K,\mathrm{inter}}\left(t\right)\;$$

Notice that photon absorption always results in an interband transition. We remark that the δ*n*_e_ depends on interband phonon emission rate only, while the *T*_e_ depends an all phonon emissions: this is obviously because all phonon emissions reduce energy but only interband phonon emissions reduce the δ*n*_e_.

Finally, the rate equations for the phonon occupation are easily obtained by requiring consistency with Equations (10) and (11) in terms of energy and particle balance. Typical results of the integration of these rate equations are reported in the Section S5 (Supporting Information).

## 5 Discussion

When a laser pulse is incident on a graphene flake, *T*_e_ increases over the pulse duration (see Section S5, Supporting Information) until it reaches a steady‐state condition. In ref. [8] we safely used a steady‐state condition in order to attribute the changes in TH signal to a single value of *T*_e_ for a fixed value of *E*_F_. This holds as long as one pulse measurement is performed on the graphene. In order to dedicate a single value to *T*_e_, either an instantaneous value or a value after the relaxation of the electrons (ps range) must be considered. However, considering the pulse durations used in our study of 110 to 150 fs, limits us from both considerations. So this intermediate state in terms of pulse duration enables us to estimate a minimum and maximum *T*_e_ for the experimental values in Figure 3. At *E*_F_ = 50 meV and CB fluence of 11 and 200 µJ cm^−2^, we estimate a *T*_e_ in the range ≈1500 to 1900 K and ≈2300 to 2500 K, respectively. At a higher value of doping (*E*_F_ = 300 meV), we estimate a *T*_e_ in the range ≈800 to 1300 K for the CB fluence of 11 µJ cm^−2^ and *T*_e_ ≈ 2200 to 2300 K for the CB fluence of 200 µJ cm^−2^.

Furthermore, the origin of the TH enhancement reported in Figure 1 resulting from a reduction in *T*_L_ can be attributed to two coherent and incoherent physical processes. First, spectral broadening induced by FB leads to band broadening and alters carrier lifetimes, thereby affecting the THGE. Second, the well‐established thermodynamics of carriers involving relaxation of carriers through optical phonons, which is temperature‐dependent, contribute to the change in THGE. In other words, the significant impact of *T*_*L*_ on the TH modulation can be qualitatively understood based on two mechanisms, which include the dependence of electronic spectral broadening Γ_*e*_ and kinetic relaxation rates *R*(*t*) on lattice temperature *T*_*L*_. The temperature dependence of Γ_*e*_ predominantly originates from the scattering of electrons by acoustic phonons, while kinetic rates depend on temperature due to the electron‐optical phonon interaction.

Finally, it is worth highlighting the interplay between the *T*_*e*_ and photoexcited enhanced Pauli blocking. Steady‐state theoretical considerations in ref. [8] predict that at low values of doping (when *E*_F_ < ℏω/2), increasing *T*_e_ will lead to the enhancement of the THG signal, a result that we were never able to observe experimentally in this work. However, these steady‐state predictions rely on the assumption that δ*n*_e_ remains constant once graphene is irradiated with a pulsed laser. In contrast, Figure 4 shows how the evolution of the TH‐MD is accompanied by both the *T*_e_ and δ*n*_e_ changes, both quantities that play a key role in the presence of both FB and CB, as discussed above. Thus, for instance, Figure 4a shows the evolution of TH‐MD when *E*_F_/(ℏω_FB_) is 0.16. For lower values of doping (corresponding to *T*_e_ ≈ 1500 to 2500 K in our experiments) and (δ*n*_e_ < 10^12^cm^2^), TH‐MD is always negative. This indicates that δ*n*_e_ is not large enough to compete with the high *T*_e_, which is consistent with the experimental observations in Figure 3. On the other hand, when *E*_F_/(ℏω_FB_) is 0.62, negative TH‐MD occurs for *T*_e_ > 1300 K (Figure 4b). Considering the *T*_e_ that we reach during the experiments (1500 to 1900 K) at this regime of doping, TH‐MD is still negative. This also confirms that δ*n*_e_ in our experiments never reaches more than 10^12^cm^2^, where TH‐MD would turn positive. It is worth mentioning that by comparing Figure 4a,b, one can immediately notice that the change in TH‐MD as a function of *T*_e_ is smaller when *E*_F_/(ℏω_FB_) is 0.62. This behavior is consistent with the results in ref. [9]. Therefore THG in graphene is always accompanied by the two competing and interconnected effects of *T*_e_ (hot electrons) and δ*n*_e_ (Pauli blocking).

To conclude, it is worth mentioning that our results could be readily applied to other gapless systems such as bilayer graphene^[^ ^42^ ^]^ and surface states of topological insulators.^[^ ^43^ ^]^ Furthermore, this work provides an interesting benchmark for the tuning of the optical nonlinearities in quantum confined systems. For instance, thermal, electrical and all‐optical tuning of harmonic generation has been widely studied in transition metal dichalcogenides (TMDs), where, however, the modulation mechanism has a completely different physical origin compared to graphene. In the case of thermal modulation, Khan et al.^[^ ^44^ ^]^ reported an enhancement of the second harmonic (SH) intensity in a MoSe_2_ monolayer of ≈25% when tuning the lattice temperature from ≈153 to 393 K and for a non‐resonant excitation wavelength of 900 nm. This SH modulation was attributed to a thermal expansion of the lattice (i.e., changes in the distance between atoms), and thus completely different with respect to the thermal modulation of graphene TH reported in this work, which we attribute to changes in the electronic distribution. Also in the case of electrical modulation, the physical mechanisms at play are completely different between graphene and TMDs. In graphene, the external gate voltage enables tuning of the Fermi energy across multi‐photon resonances in the Dirac cone.^[^ ^8^, ^21^ ^]^ In contrast, electrical modulation in TMDs is mainly due to tuning of the optical resonances from neutral to charged excitons (trions).^[^ ^6^ ^]^ Furthermore, gate tuning also leads to different recombination dynamics both in graphene and TMDs. In TMDs, such changes are attributed to the different lifetime of trions with respect to neutral excitons.^[^ ^45^ ^]^ In contrast, in graphene the gate tuneable recombination dynamics are due to quenching of the scattering between hot electrons and optical phonons.^[^ ^38^ ^]^ For the all‐optical modulation of harmonic generation in TMDs, we have recently demonstrated a modulation scheme for SHG^[^ ^4^ ^]^ which fully exploits the crystal symmetry and it allows to rotate the polarization of the emitted SH signal by 90° on ultrafast timescales. This ultrafast SH tuning can be exploited, in combination with specifically designed metasurfaces, to achieve light wavefront shaping.^[^ ^46^ ^]^ A second scheme for all‐optical modulation exploits Pauli blocking. This is possible both in graphene, as demonstrated in this work, and in TMDs.^[^ ^7^ ^]^ Here, the main difference is that all‐optical modulation by Pauli blocking can be realized in principle at any wavelength in graphene, due to its linear band absorption and broadband absorption, while in TMDs all‐optical modulation of the NLO response is efficient only at resonance with excitonic transitions.^[^ ^47^ ^]^

## 6 Conclusion

In conclusion, we performed a detailed experimental and theoretical study of static thermal and ultrafast opto‐electronic modulation of THG in a high‐quality graphene FET encapsulated in thin hBN layers. We have demonstrated static switching of THG via tuning of the lattice temperature and electron doping, with a peculiar ambipolar behavior that arises from the electron‐hole symmetry in the Dirac cone, and a factor of ≈1.5 modulation of the *P*_*TH*_ at *E*_F_ = 50 meV and of ≈3 at *E*_F_ = 300 meV when tuning the *T*_L_ from room temperature to 33 K. We suggest that this result originates from the spectral relaxation and thermodynamic kinetics of carriers. Furthermore, we have established all‐optical ultrafast control of graphene THG with gate tunable dynamics, and achieved up to 85% ultrafast opto‐electronic modulation depth of the TH at *E*_F_ = 300 meV and fluence of 200 µJ cm^−2^, which is two orders of magnitude more efficient compared to previous reports. We discuss the *E*_F_ dependent temporal dynamics of all‐optical TH modulation due to quenching of the phase‐space scattering between optical phonons and electrons.^[^ ^30^ ^]^ This provides a powerful tool to actively control both the TH modulation depth and the recombination dynamics in graphene opto‐electronic nonlinear devices. Finally, we have addressed these experimental observations with a detailed theoretical framework that explains the ultrafast opto‐electronic modulation of TH in graphene to be rooted in a mixed effect of Pauli blocking and carrier electronic temperature. Consequently, our work can be seen as the first step toward a holistic approach for manipulating not only THG, but more in general any third‐order non‐linear optical effect in graphene, such as saturable absorption for synchronized dual‐fiber lasers^[^ ^48^ ^]^ or THz pulse generation.^[^ ^49^ ^]^ Furthermore, our findings can be used to optimize the performances of nonlinear optical devices for applications in gas sensing^[^ ^11^ ^]^ and logic gates,^[^ ^12^ ^]^ and for the development of hybrid photonic devices for the enhancement of optical nonlinearities, for instance in fiber‐^[^ ^50^ ^]^ and waveguide‐based frequency converters.^[^ ^51^ ^]^ Thus, thanks to a detailed description of the transient nonlinear optical, electronic, and thermal response of graphene, this work will be relevant for the design of a wide range of nanoscale and ultrafast nonlinear optical devices.

## Conflict of Interest

The authors declare no conflict of interest.

## Supporting information

Supporting Information

## Data Availability

The data that support the findings of this study are available from the corresponding author upon reasonable request.
