# Supplementary material for: Ultrafast Opto‐Electronic and Thermal Tuning of Third‐Harmonic Generation in a Graphene Field Effect Transistor
Source: Adv Sci (Weinh). 2024 Jun 18;11(31):2401840. doi: 10.1002/advs.202401840 (PMC11336917; doi:10.1002/advs.202401840)
Supplement: Supplementary file 1 — Supporting Information [file ADVS-11-2401840-s001.pdf]

ADVANCED SCIENCE
Open Access

Supporting Information

for *Adv. Sci.*, DOI 10.1002/advs.202401840

Ultrafast Opto-Electronic and Thermal Tuning of Third-Harmonic Generation in a Graphene Field Effect Transistor

*Omid Ghaebi, Sebastian Klimmer, Nele Tornow, Niels Buijssen, Takashi Taniguchi, Kenji Watanabe, Andrea Tomadin, Habib Rostami and Giancarlo Soavi**

SUPPORTING INFORMATION

Ultrafast Opto-Electronic and Thermal Tuning of Third-Harmonic Generation in a Graphene Field Effect Transistor

AUTHOR LIST

Omid Ghaebi[1], Sebastian Klimmer[1,2], Nele Tornow[1], Niels Buijssen[1], Takashi Taniguchi[3], Kenji Watanabe[4], Andrea Tomadin[5], Habib Rostami[6], Giancarlo Soavi[1,7]

AFFILIATIONS

[1]Institute of Solid State Physics, Friedrich Schiller University Jena, Jena, 07743, Germany
[2]ARC Centre of Excellence for Transformative Meta-Optical Systems, Department of Electronic Materials Engineering, Research School of Physics, The Australian National University, Canberra, ACT 2601, Australia
[3]Research Center for Materials Nanoarchitectonics, National Institute for Materials Science, 1-1 Namiki, Tsukuba 305-0044, Japan
[4]Research Center for Electronic and Optical Materials, National Institute for Materials Science, 1-1 Namiki, Tsukuba 305-0044, Japan
[5]Dipartimento di Fisica, Università di Pisa, Largo Bruno Pontecorvo 3, 56127 Pisa, Italy
[6]Department of Physics, University of Bath, Claverton Down, Bath BA2 7AY, UK
[7]Abbe Center of Photonics, Friedrich Schiller University Jena, Jena, 07743, Germany

S1 DEVICE FABRICATION

The monolayer graphene flake was exfoliated from bulk synthetic graphite (HQ-graphene) using Scotch tape (Minitron). The hBN layers and graphite contacts were exfoliated with the same method on silicon wafers. The thickness of the hBN layers were determined by optical contrast following the approach described in Ref. [1]. A thin stamp comprising PC (polycarbonate) on a glass slide was prepared [2] an subsequently used to pick up the hBN layers, graphite contacts, and graphene using a commercial transfer stage (HQ-graphene).

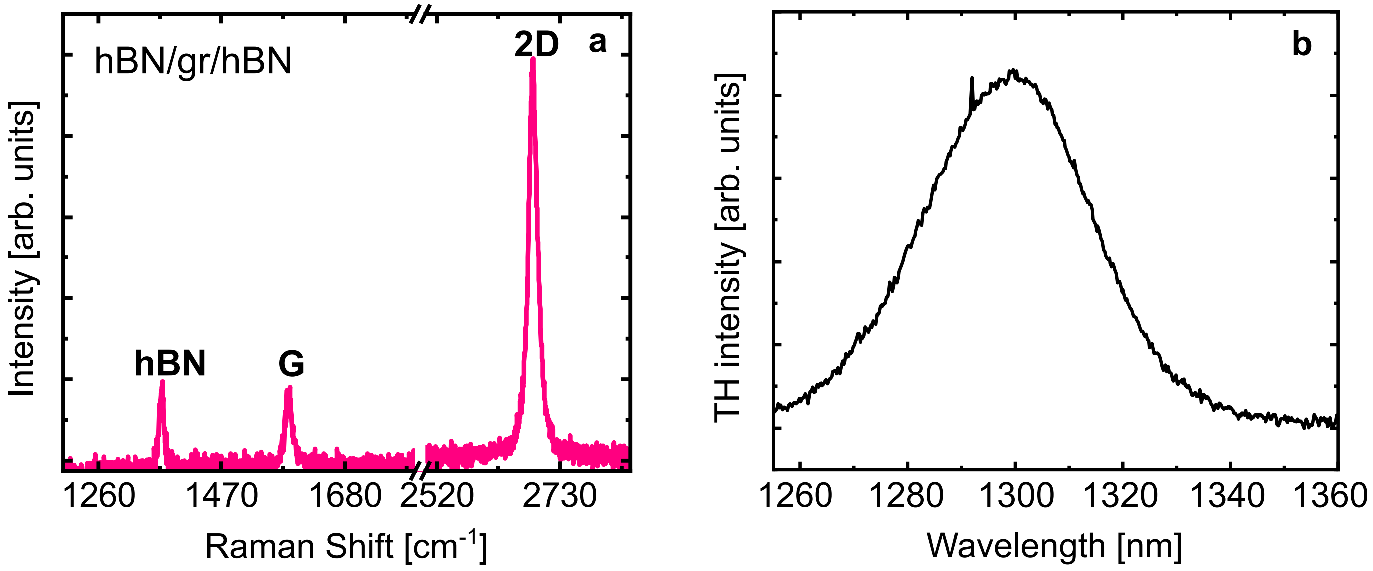

Fig. S1. **Raman characterization and TH spectrum. a)** Raman spectrum of the device showing the 2D peak of graphene at 2683 cm^{-1}, the G peak of graphene at 1596 cm^{-1} and the E_{2g} mode of hBN located at 1368 cm^{-1}. **b)** TH spectrum of the FB.

Subsequently, the layers were transferred to a silicon wafer (90 nm SiO_2) with pre-patterned gold contacts.

S2 RAMAN CHARACTERIZATION AND CARRIER MOBILITY ESTIMATION

After fabrication, we characterized the device with Raman spectroscopy. The Raman spectrum of the sample after hBN encapsulation is depicted in Fig. S1a. The 2D peak at 2683 cm^{-1} is a single Lorentzian, confirming the monolayer nature of our sample [3]. The low FWHM (Full Width at Half Maximum) of the G and 2D peaks (FWHM(G) = 13.5 cm^{-1}, FWHM(2D) = 17.7 cm^{-1} indicates a negligible strain of the graphene flake after transfer [4]. The peak at 1368 cm^{-1} belongs to the hBN bottom and top flakes (in-plane atom vibrations)[5, 6]. The absence of the D peak of graphene, typically at 1350 cm^{-1}, is a further indication of the high quality of our sample [7–9].

Further, we estimated the mobility of the device from the black $I_{SD} - V_G$ curve in Fig. 1b of the main text. The carrier mobility is proportional to the first derivative of I_{SD} with

respect to V_{G} via equation (1) [2, 10]:

$$\mu = \frac{1}{c_{\mathrm{eff}}}\frac{d\sigma}{dV_{\mathrm{G}}} = \frac{L}{\mathrm{w}\epsilon}\ \frac{d_c}{v_{\mathrm{SD}}}\frac{dI_{\mathrm{SD}}}{dV_{\mathrm{G}}} \tag{1}$$

where L and w define the size of the channel (graphene flake), ϵ is the relative permittivity, and c_{eff} is the effective capacitance of the dielectric spacer (hBN/SiO_2). The thickness of hBN is $\sim 10\,\mathrm{nm}$ (identified by color based on Ref.[1]) and the oxide thickness is 90 nm. Therefore, c_{eff} can be calculated considering hBN and oxide layers in series [2]. From this, we obtained a mobility of $\sim 25\,000\,\mathrm{cm}^2/\mathrm{Vs}$.

In a graphene FET, V_{G} creates an electrostatic potential between graphene and the gate electrode which can tune the E_{F} by changing the electron density n_{e}. As a result, V_{G} is given by :

$$V_{\mathrm{G}} = \frac{E_{\mathrm{F}}}{e} + \varphi \tag{2}$$

The first and second terms are determined by the quantum and geometrical (effective) capacitance (c_{eff}), respectively. For a back-gated sample, the geometrical capacitance dominates over the quantum capacitance: $V_{\mathrm{G}} \sim \varphi = \frac{n_{\mathrm{e}}e}{c_{\mathrm{eff}}}$ [11]. In graphene, the E_{F} is proportional to the square root of n_e *via* the relation $E_{\mathrm{F}} = \hbar v_{\mathrm{F}}\sqrt{n_{\mathrm{e}}}$ with $v_{\mathrm{F}} \sim 10^6\,\mathrm{m/s}$ [12]. Considering $c_{\mathrm{eff}} = \frac{\epsilon_{\mathrm{r}}\epsilon_0}{d_c}$, E_{F} can be written as:

$$E_{\mathrm{F}} = \hbar v_{\mathrm{F}}\sqrt{\frac{\pi\epsilon_0\epsilon_{\mathrm{r}}(V_{\mathrm{G}} - V_{\mathrm{CNP}})}{ed_c}} \tag{3}$$

where ϵ_{r} and d_c are the relative permittivity and thickness of the capacitor and V_{CNP} is the gate voltage at minimum conductance (Charge Neutrality Point, CNP).

To ensure that the signal measured in our experiments is the TH of the FB, we recorded its spectrum (Fig. S1b) using a spectrometer consisting of a monochromator (Horiba iHR 320) and a liquid nitrogen cooled InGaAs detector (Horiba Symphony II). By fitting the spectrum with a Gaussian function, we obtain a central wavelength of $\sim$ 1300 ($\pm$ 0,02) nm, namely $\lambda_0/3$, where λ_0 is the FB wavelength, as expected for a THG process.

S3 THG EXPERIMENTAL SETUP

The CB and FB pulses were obtained from a Yb-based femtosecond oscillator (FLINT12, Light Conversion) and OPO (Levante fs IR, APE), respectively. The main laser source

(FLINT12 from Light Conversion), is a mode-locked Yb laser operating at a wavelength of 1030 nm, repetition rate of 76 MHz and 12 W average power. A portion of this (4.5 W) is used to pump the OPO, which provides tunable output in the range 1320 to 2000 nm for the signal and 2150 to 4800 nm for the idler. The latter was used as the FB in our experiments. The pulse duration of FB and CB are 150 and 110 fs, respectively. The relative delay between CB and FB is controlled by a motorized delay line (M-404.2PD, PI). The two pulses are subsequently combined on a beam splitter (BS), after which they propagate collinearly into a home-built microscope and they are finally simultaneously focused on the sample with a spot size of $\sim 6.7\,\mu$m (FB) and $\sim 2.2\,\mu$m (CB), measured from the razor blade technique [13]. In all the experiments, the sample was mounted inside an optical cryostat (ST-500, Janis) coupled to a silicon temperature controller (Lakeshore) integrated with built-in stages (Attocube, ANPX101/LT and ANPZ102/RES/LT). The backward emitted TH signal is spectrally filtered and detected on an amplified InGaAs photoreceiver (model 2153, Newport). The TH signal experiences a total loss of 92.8% in propagation through different optical components and considering the quantum efficiency of the detector. The peak fluence of the FB is kept at $\sim 130\,\mu\text{J/cm}^2$ for static and at $\sim 110\,\mu\text{J/cm}^2$ for all-optical modulation THG experiments, respectively, while the CB peak fluence is tuned in the range 11 to $200\,\mu\text{J/cm}^2$.

S4 THIRD HARMONIC GENERATION FROM THE hBN ENCAPSULANT

Graphene has a strong nonlinear response thanks to its linear band dispersion that allows gate-tunable resonant light-matter interactions [14] at almost any wavelength in the visible and near/mid-IR spectral region. In contrast, hBN is a wide gap ($\approx$ 6 eV) dielectric material and THG is always non-resonant (and thus negligible) for the photon energies of our experiments. To confirm this hypothesis, we first provide an estimate of the hBN TH average power based on published results. For instance, Popkova *et al.* [15] measured $\approx$ 2.5 pW of TH average power for a $\approx$ 20 nm hBN flake at an excitation wavelength of 1080 nm and $0.01\,\text{J/cm}^2$ of incident peak fluence. Assuming a dispersionless nonlinear response up to 0.32 eV (*i.e.*, the excitation photon energy in our experiments), we can estimate the expected average TH power under our experimental conditions. Thus, for the same hBN thickness of $\approx$ 20 nm and considering our incident peak fluence of $\approx 130\,\mu\text{J/cm}^2$, we expect much less

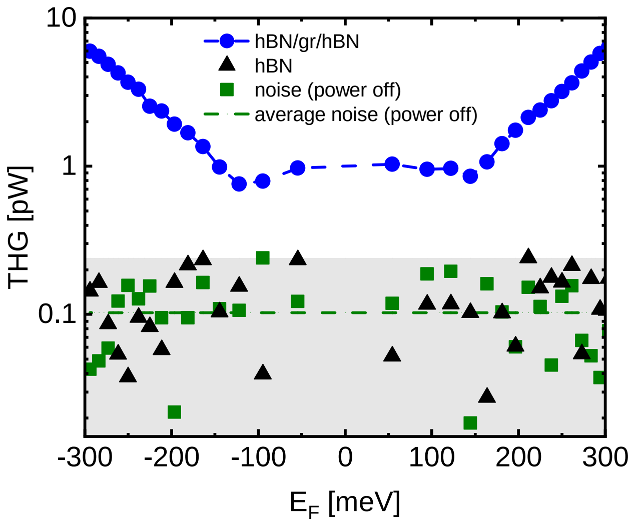

Fig. S2. TH average power as a function of E_F on hBN/gr/hBN (blue circles) and on a $\approx$ 20 nm thick hBN (black triangles) at a lattice temperature of T_L=33 K. The grey shaded area represents the noise floor of our detector.

than 1 fW ($\approx$ 5 attoW) of average TH power, a value that is well below the sensitivity of our setup ($\approx$ 0.1 pW). To further substantiate this estimate, we performed gate dependent TH experiments in a region of the sample without graphene, where only the hBN (with a thickness of $\approx$ 20 nm) is present. The data are shown in Fig. S2, which shows that the emitted TH power from hBN falls within the noise floor of our detector (model 2153, Newport fW receiver) given our experimental conditions. Note that the noise floor is measured as the "light-off" condition (*i.e.*, when we don't illuminate the sample with the fundamental beam) and thus it really represents the ultimate limit to the detection of a TH signal.

S5 TIME-EVOLUTION OF THE ELECTRON DISTRIBUTION

Fig. S3 shows the time-evolution of electron thermodynamic variables for a set of typical parameters. With reference to the model described in the main text, we use a typical integration timestep t $\sim$ 5 fs and refractive indices of the SiO_2 substrate n_t, n_b = 1.4, ignoring the thin hBN layers because excitations do not overlap with the frequency ranges where the material features hyperbolic dispersion. The phonon rates $R_{\Gamma,K}$, given *e.g.* in Ref. [16] are proportional to the electron-phonon coupling parameter $\partial t/\partial b$, see also Refs. [17, 18]. We use the value $\partial t/\partial b = 200\,\text{eV/nm}$, substantially larger than found in the literature,

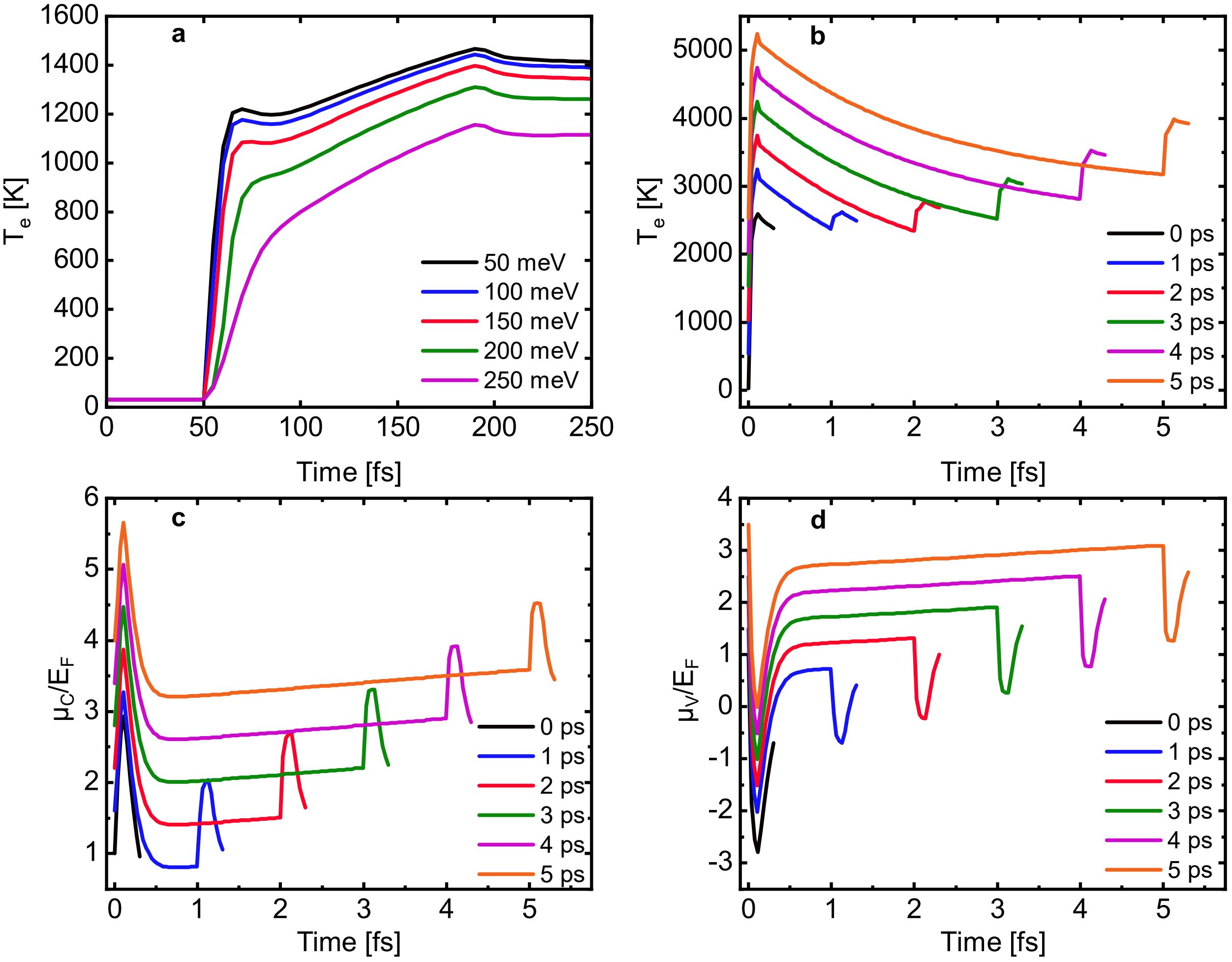

Fig. S3. **Time-evolution of T_{e}, μ_{C}, and μ_{V}.** Time-evolution of T_{e} with **a** a single pulse and several values of E_{F} and with **b** two pulses as the delay is increased. **c,d** Time-evolution of $\mu_{\mathrm{C}}/E_{\mathrm{F}}$ and $\mu_{\mathrm{V}}/E_{\mathrm{F}}$ **d** for the same conditions as **b**. The plot legends indicate the time delay between FB and CB pulses.

to address at a phenomenological level the enhanced electron recombination that has been reported in Refs. [16, 19].

In (a) the electron temperature is shown for several values of E_F, in the absence of a CB, under a FB active from $t = 50$ fs, of duration 150 fs and fluence 130 µJ/cm^2. Temperatures of the order of 10^3 K are achieved during the FB. As expected, a higher E_F leads to lower T_e, because a higher carrier density entails a larger heat capacity.

Panels (b)-(d) correspond to a dynamics including a CB active from $t = 0$, of duration 110 fs and fluence 200 µJ/cm^2, and a FB of duration 150 fs and fluence 110 µJ/cm^2, active

after an initial delay shown in the legend. The curves are displaced along the vertical axis for visibility.

In particular, (b) shows that a FB with same fluence leads to markedly different final temperature, based on the initial condition of the electron system. One also has to keep in mind that the absorption coefficient in graphene does depend on temperature and the chemical potentials, and thus changes with the delay. It is also important to notice that T_e is not constant during the FB, even if a smaller variation is experienced compared to case (a), where the electron system is in equilibrium at the lattice temperature of 30 K before the FB. Finally, the role of the photoexcited density is clearly visible in (d), where a change of sign of the chemical potential in the valence band takes place during the CB and FB, corresponding to a large quantity of holes being left behind by the electrons promoted to the conduction band.

We reiterate that all these results are obtained assuming the quasi-equilibrium form (equation (9) of the main text) for the electron distribution, and can only be understood in the sense of a coarse-grained representation of the time-evolution, on a time-step larger than the thermalization time-scale of $\sim 20\,\mathrm{fs}$.

S6 FINITE CONDUCTIVITY AND THE ELECTRON SCATTERING RATE

The electron scattering rate Γ_e that enters equation (6) of the main text can be expressed as the sum of scattering rates from different sources: $\Gamma_e = (\Gamma_e)_\mathrm{ac} + (\Gamma_e)_\mathrm{imp}$. Refs. [20–22] provide formulas for the electron scattering rate due to scattering from long-range charged impurities and short-range disorder, given by $(\Gamma_e)_\mathrm{imp} = (\Gamma_e)_\mathrm{long} + (\Gamma_e)_\mathrm{short}$:

$$(\Gamma_e)_\mathrm{long} \approx \frac{n_\mathrm{i}(\pi r_\mathrm{s})^2}{2} \left\{ \frac{(\hbar v_\mathrm{F})^2}{|E_\mathrm{F}|} \right\}, \tag{4}$$

$$(\Gamma_e)_\mathrm{short} \approx \frac{n_\mathrm{d} V_0^2}{8} \left\{ \frac{|E_\mathrm{F}|}{(\hbar v_\mathrm{F})^2} \right\}, \tag{5}$$

where n_i is charged impurity center density, $r_\mathrm{s} = e^2/(\hbar v_\mathrm{F} \kappa)$ with κ being the dielectric constant, n_d is the short-range impurity density and V_0 is a constant short-range potential strength. For low $T_\mathrm{L} < 200\,\mathrm{K}$, the resistivity of graphene is primarily influenced by scattering with acoustic phonons, contributing to the electron scattering rate at the Fermi surface as

follows [23]

$$(\Gamma_e)_{\rm ac} \approx \frac{D^2}{8\rho_m v_{\rm s}^2} \left\{ \frac{|E_{\rm F}|}{(\hbar v_{\rm F})^2} \right\} (k_B T_L), \tag{6}$$

where $D = 19\,{\rm eV}$ is the deformation potential, $v_{\rm s} \sim 2 \times 10^6\,{\rm cm/s}$ is the sound velocity, and $\rho_{\rm m} = 7.6 \times 10^{-8}\,{\rm g/cm^2}$ is the mass density.

Taking into account contributions from acoustic phonons, long-range charged impurities, and short-range disorder, we can use the following empirical ansatz for the electron scattering rate:

$$\Gamma_{\rm e} = A|E_{\rm F}| + \frac{B}{|E_{\rm F}|} + C|E_{\rm F}|(k_B T_L), \tag{7}$$

where A, B, and C are empirical parameters and in quasi-equilibrium condition we can replace E_F with the average of chemical potential in the conduction and valence bands: $E_{\rm F} \to (\mu_{\rm C} + \mu_{\rm V})/2$. By relating the electron scattering rate to the mobility and fitting the mobility *vs.* E_F to experimental data we find $B = 0.0013\,{\rm eV}^2$. At present, we neglect the coefficients A and C for simplicity.

---

[1] Anzai, Y. *et al.* Broad range thickness identification of hexagonal boron nitride by colors. *Appl. Phys. Express* **12**, 055007 (2019).

[2] Purdie, D. *et al.* Cleaning interfaces in layered materials heterostructures. *Nat. Commun.* **9**, 1–12 (2018).

[3] Ferrari, A. C. *et al.* Raman spectrum of graphene and graphene layers. *Phys. Rev. Lett.* **97**, 187401 (2006).

[4] Neumann, C. *et al.* Raman spectroscopy as probe of nanometre-scale strain variations in graphene. *Nat. Commun.* **6**, 1–7 (2015).

[5] Schué, L., Stenger, I., Fossard, F., Loiseau, A. & Barjon, J. Characterization methods dedicated to nanometer-thick hBN layers. *2D Mater.* **4**, 015028 (2016).

[6] Geick, R., Perry, C. & Rupprecht, G. Normal modes in hexagonal boron nitride. *Phys. Rev.* **146**, 543 (1966).

[7] Tuinstra, F. & Koenig, J. L. Raman spectrum of graphite. *J. Chem. Phys.* **53**, 1126–1130 (1970).

[8] Ferrari, A. C. & Robertson, J. Interpretation of raman spectra of disordered and amorphous carbon. *Phys. Rev. B* **61**, 14095 (2000).

[9] Ferrari, A. C. & Basko, D. M. Raman spectroscopy as a versatile tool for studying the properties of graphene. *Nat. Nanotechnol.* **8**, 235–246 (2013).

[10] De Fazio, D. *et al.* High-mobility, wet-transferred graphene grown by chemical vapor deposition. *ACS Nano* **13**, 8926–8935 (2019).

[11] Das, A. *et al.* Monitoring dopants by raman scattering in an electrochemically top-gated graphene transistor. *Nat. Nanotechnol.* **3**, 210–215 (2008).

[12] Novoselov, K. S. *et al.* Two-dimensional gas of massless dirac fermions in graphene. *Nature* **438**, 197–200 (2005).

[13] Kimura, S. & Munakata, C. Method for measuring the spot size of a laser beam using a boundary-diffraction wave. *Opt. Lett.* **12**, 552–554 (1987).

[14] Soavi, G. *et al.* Broadband, electrically tunable third-harmonic generation in graphene. *Nat. Nanotechnol.* **13**, 583–588 (2018).

[15] Popkova, A. A. *et al.* Optical third-harmonic generation in hexagonal boron nitride thin films. *ACS Photonics* **8**, 824–831 (2021).

[16] Pogna, E. A. A. *et al.* Electrically tunable nonequilibrium optical response of graphene. *ACS Nano* **16**, 3613–3624 (2022). URL `https://doi.org/10.1021/acsnano.1c04937`.

[17] Rana, F. *et al.* Carrier recombination and generation rates for intravalley and intervalley phonon scattering in graphene. *Phys. Rev. B* **79**, 115447 (2009).

[18] Wang, H. *et al.* Ultrafast relaxation dynamics of hot optical phonons in graphene. *Appl. Phys. Lett.* **96** (2010).

[19] Pogna, E. A. *et al.* Hot-carrier cooling in high-quality graphene is intrinsically limited by optical phonons. *ACS Nano* **15**, 11285–11295 (2021).

[20] Shon, N. H. & Ando, T. Quantum transport in two-dimensional graphite system. *J. Phys. Soc. Jpn.* **67**, 2421–2429 (1998). URL `https://doi.org/10.1143/JPSJ.67.2421`.

[21] Ando, T. Screening effect and impurity scattering in monolayer graphene. *J. Phys. Soc. Jpn.* **75**, 074716 (2006). URL `https://doi.org/10.1143/JPSJ.75.074716`.

[22] Das Sarma, S., Adam, S., Hwang, E. H. & Rossi, E. Electronic transport in two-dimensional graphene. *Rev. Mod. Phys.* **83**, 407–470 (2011). URL `https://link.aps.org/doi/10.1103/RevModPhys.83.407`.

[23] Hwang, E. H. & Das Sarma, S. Acoustic phonon scattering limited carrier mobility in two-dimensional extrinsic graphene. *Phys. Rev. B* **77**, 115449 (2008). URL `https://link.aps.org/doi/10.1103/PhysRevB.77.115449`.
